# Callus induction and transcriptomic analysis of *in vitro* embryos at different developmental stages of peony

**DOI:** 10.3389/fpls.2022.1046881

**Published:** 2022-11-03

**Authors:** Xiangtao Zhu, Huijun Zhu, Wen Ji, Erman Hong, Zeyun Lu, Bole Li, Xia Chen

**Affiliations:** ^1^ College of Jiyang, Zhejiang A&F University, Zhuji, China; ^2^ Zhejiang Provincial Key Laboratory of Germplasm Innovation and Utilization for Garden Plants, Zhejiang A&F University, Hangzhou, China

**Keywords:** ‘Fengdanbai’, embryo *in vitro*, callus induction, plant growth regulator, transcriptome

## Abstract

The efficient induction of peony embryogenic callus is of great significance to the improvement and establishment of its regeneration technology system. In this study, the *in vitro* embryos of ‘Fengdanbai’ at different developmental stages were selected as explants, the effects of different concentrations and types of plant growth regulator combinations on the induction and proliferation of embryonic callus at different developmental stages were investigated, and comparative transcriptome analysis of callus with different differentiation potentials were performed to explore the molecular mechanisms affecting callus differentiation. The results showed that the germination rate of 90d seed embryo was the best, which was 94.17%; the 70d and 80d cotyledon callus induction effect was the best, both reaching 100%, but the 80d callus proliferation rate was higher, the proliferation rate reached 5.31, and the optimal induction medium was MS+0.1 mg·L^–1^NAA+0.3 mg·L^–1^TDZ+3 mg·L^–1^2,4-D, the callus proliferation multiple was 4.77. Based on the comparative transcriptomic analysis, we identified 3470 differentially expressed genes (DEGs) in the callus with high differentiation rate and low differentiation rate, including 1767 up-regulated genes and 1703 down-regulated genes. Pathway enrichment analysis showed that the “Phenylpropanoid biosynthesis” metabolic pathway was significantly enriched, which is associated with promoting further development of callus shoots and roots. This study can provide reference for genetic improvement and the improvement of regeneration technology system of peony.

## 1 Introduction

Peony (*Paeonia suffruticosa* Andr.) is a perennial deciduous shrub of the *Paeonia* family. Peony is one of the traditional famous flowers in my country, known as the “king of flowers” (Zhang et al., 2017). In recent years, peony has developed in the direction of oil use and cut flowers, which has great economic value and development potential (Hu, 2015). The propagation of traditional peony is mainly based on seed propagation, cuttings, grafting and ramets, but seeds have dormancy characteristics, with low rooting rate and germination rate, and ramet propagation has the problem of weak reproductive ability, cuttings and grafting are greatly affected by the environmental season, the reproduction scale is small and the speed is slow, and it is difficult to mass produce, which seriously restricts the large-scale development of the peony industry and cannot meet people’s increasing demand for peony (Wen et al., 2016; Wen et al., 2020). Especially for seed propagation, it takes at least 1-2 years from the formation of seedlings to stable flowering. If the process of primary selection and re-election is carried out, it will take at least 10 years to launch a new variety (Cheng and Du, 2008). In addition, in the process of cross-breeding, the development of seed embryos is incomplete or even stopped, which seriously hinders the breeding process. The germination of seed embryos is of great significance for embryo rescue to overcome the incompatibility problem of distant crosses (Gu et al., 2010). Therefore, in order to meet the market demand and speed up the process of peony breeding, embryo rescue technology has been widely used in breeding work (He, 2006; Silva et al., 2012). Previous studies have shown that through the cultivation of immature embryos, the hybrid embryos that have been aborted by distant hybridization of plants can be successfully rescued, thus creating the application of embryo culture technology in distant hybridization breeding, at the same time, the regeneration of seedlings by culturing zygotic embryos has the characteristics of small variation, short cycle and simple operation (Wang et al, 2002; Raghavan, 2003; Gregory, 2004).

As an important parent of peony breeding, “Fengdanbai” has the characteristics of abundant pollen grains, high seed setting rate, fast growth rate and strong stress resistance. Therefore, it is of great significance to study the germination and callus induction, proliferation and differentiation of seed embryos of Fengdanbai at different developmental stages for embryo rescue (Ren et al., 2020). The research on the rescue of peony seed embryos mainly includes the *in vitro* culture research of peony seed embryos. Previous studies have shown that light conditions, explant disinfection time, hormone concentration ratio, basic medium, and seed embryo maturity are important for the *in vitro* culture of peony seed embryos (Chang et al., 2019; Lu et al., 2019; Liu et al., 2021), in the selection of seed embryo maturity, the germination rate of young embryos was significantly higher than that of mature embryos (Chang et al., 2019; Lu et al., 2019; Lu et al., 2021), and in the subsequent growth, adding NAA, IAA, 6-BA, GA3, TDZ, PUT and other substances can improve the expansion rate, germination rate and uniformity of seed embryos (He et al., 2019; Wang, 2020; Liu et al., 2021). Compared to other explants, the seed embryo has the best physiological state, higher regeneration potential and the lowest contamination rate after inoculation, which is an ideal material for inducing callus of peony (Lu et al., 2019). Studies on the induction of callus in peony seed embryos have shown that the suitable hormones for callus induction are NAA, 2-4D and 6-BA (Xu et al., 2017; Wang, 2020; Liu et al., 2022), in which immature embryos are the most suitable material for callus induction (Yin et al., 2013; Liu et al., 2022), it is easier to obtain high-quality callus by completely breaking the cut immature embryo slices in the cutting method (Liu et al., 2022). The study of callus proliferation process showed that the pre-culture time, light quality, pH value, agar concentration, sucrose concentration and subculture period of explants all had certain effects on the proliferation of callus (Chen, 2013; Cheng et al., 2019; Wang et al., 2020), and GA_3_, NAA, and hydrolyzed casein (CH) were beneficial to promote the proliferation of callus and inhibit its browning (Zhang and Tang, 2015; Cheng et al., 2019; Wang et al., 2020). Due to the immaturity of the callus differentiation system of peony seed embryos, there are few relevant research reports, which is also an important reason for restricting the establishment of peony regeneration system. The differentiation ability of tissue is related to plant material, medium, type and concentration of plant growth regulators, etc. Among them, 2,4-D is not conducive to the differentiation of ‘Fengdanbai’ peony embryo callus adventitious buds (Lu et al., 2021), while H_2_O_2_, TDZ, 2,4-D, 6-BA, KT, ZT and other substances can promote the differentiation of callus (He et al., 2015; Zhang and Tang, 2015; Lu et al., 2019; Wang et al., 2020). In the research on the mechanism of callus differentiation, predecessors mainly focused on the physiological and biochemical changes of callus differentiation, for example, Wang et al. (2010) found that the expression of acidic proteins changed significantly during rooting induction; the content of paeonol and the activities of POD, SOD, CAT and other substances changed significantly during the growth of callus (Ji, 2019). Although the current molecular mechanism of callus differentiation has been reported in other plants (Zhang and Deng, 2018; Xu et al., 2021), there are few related studies in peony, Zhang et al. (2022) used methylation-sensitive amplified polymorphism (MSAP) technology to compare the DNA methylation of non-embryonic and embryogenic callus, unrooted and rooted tissue culture seedlings of “Fengdanbai”, it was speculated that the embryogenic callus of “Fengdanbai” was less differentiated, which may be related to the formation of hypermethylation. Jie et al. (2020) determined the key genes involved in callus browning by sequencing the transcriptome of the petiole callus of peony “Kao”. However, the different morphological differences of peony embryogenic callus affect the late differentiation potential and its molecular mechanism has not been reported yet. In this study, the seeds of “Fengdanbai” at different developmental stages were used as materials to study the effects of different concentrations and different combinations of plant growth regulators and developmental stages on callus induction, and screened out the optimal medium and optimal development period in the selected concentration. At the same time, the callus with different external morphological differences with different differentiation rates in the later stage was subjected to transcriptome sequencing, in order to lay a theoretical foundation for improving the quality of peony callus and promoting callus differentiation, and to provide a theoretical basis for exploring the molecular mechanism of callus differentiation on the basis of establishing the regeneration system of peony.

## 2 Materials and methods

### 2.1 Experimental materials

**Figure 1:**
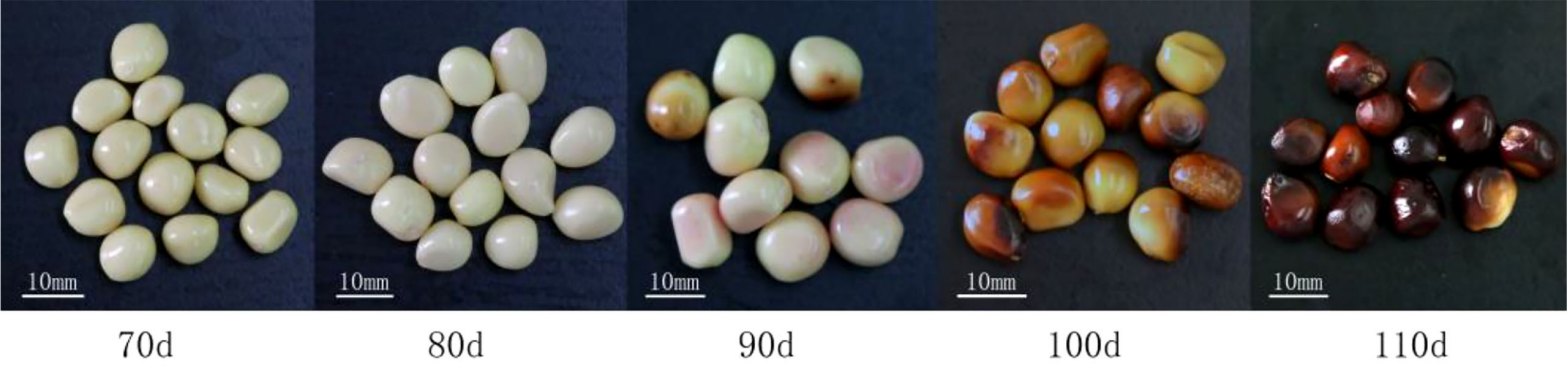
The seeds of “Fengdanbai” at different developmental stages after flowering.

The seeds of the peony ‘Fengdanbai’ at different developmental stages were used as materials, and the plants were planted in the Peony Base of Zhejiang Agriculture and Forestry University. The seeds of ‘Fengdanbai’ at different developmental stages (70d, 80d, 90d, 100d, 110d) after flowering (**Figure 1**) were peeled from the husk and put into a beaker, washed with detergent, and rinsed with running water for 2 hours. Transfer to the ultra-clean workbench, soak in 2% NaClO solution for 20min, rinse with sterile water for 3-5 times, rinsed for 10 seconds each time, then disinfect with 75% alcohol for 8-10s, rinse with sterile water for 3-5 times, and then placed on sterile filter paper to absorb water, peel off the seed embryo with a scalpel and tweezers, and inoculate it on corresponding medium for culture.

### 2.2 The effect of different developmental stages on embryo germination

*In vitro* embryos (the embryos are stripped from seeds at different stages of development) of “Fengdanbai” at different developmental stages were used as explants, inoculated on MS medium (Murashige and Skoog, 1962), cultured in the dark for 30 days, and the germination rates of “Fengdanbai” *in vitro* embryos at different developmental stages after flowering were counted to analyze the effect of different developmental stages after flowering on the germination of *in vitro* embryos. Germination rate of *in vitro* embryos = number of germinated *in vitro* embryos/total number of inoculations × 100%.

### 2.3 Effects of explants at different developmental stages on callus induction

The cotyledons (cut off and chop the cotyledons that germinate from the *in vivo* embryos) excised from the 70d, 80d, 90d, 100d, and 110d embryos of “Fengdanbai” were used as explants and inoculated into MS+NAA 0.1 mg·L^–1^+TDZ 0.3 mg·L^–1^ medium to analyze the effect of embryonic explants at different developmental stages after flowering on callus induction, and screened out the most suitable developmental period for callus induction. Induction rate = the number of callus induced/the number of inoculation × 100%; Proliferation multiples of callus = total mass of callus generated/total mass of callus at transfer.

### 2.4 Screening of the most suitable callus induction medium

The cotyledons excised after germination of 80-day embryo of “Fengdanbai” were inoculated on the callus-induced MS medium with different concentrations of NAA (0.1, 0.3, 0.5 mg·L^–1^), TDZ (0.1, 0.2, 0.3 mg·L^–1^) and 3.0 mg·L^–1^2,4-D respectively to analyze the effects of different concentrations and combinations of plant growth regulators on callus induction, and screen out the most suitable callus-induced culture medium.

### 2.5 Culture conditions

All media were supplemented with 30 g·L^–1^ sucrose, 7.0 g·L^–1^ agar, 3.0 g·L^–1^ activated carbon, pH 5.7-5.8. The culture temperature was 23 ± 3°C, the light intensity was 35-40μmol·m-^2^s^-1^, and the relative humidity was 40%-70%.In the callus induction stage, the cells were cultured in dark for 15 days and then transferred to light conditions, the light time is 8 h·d^-1^, and the light time of seed embryo germination stage is 10 h·d^-1^. Each of the above treatments was repeated 3 times with 10 vials of 4 explants per vial.

### 2.6 Transcriptome sequencing of callus with different differentiation potential

The callus with different morphological characteristics were chopped, immediately placed in liquid nitrogen and then stored in a -80°C ultra-low temperature refrigerator for future use. Three bottles of callus with two different morphological characteristics were selected as biological replicates, respectively, the callus in each bottle (3-4) was mixed.

#### 2.6.1 RNA extraction, cDNA library construction and sequencing

Total RNA was extracted using Trizol reagent kit (Invitrogen, Carlsbad, CA, USA) according to the manufacturer’s protocol. Then mRNA was enriched by Oligo(dT) beads, the enriched mRNA was fragmented into short fragments using fragmentation buffer and reverse transcribed into cDNA with random primers (NEB#7530 Kit, New England Biolabs). Second-strand cDNA was synthesized by DNA polymerase I, RNase H, dNTP and buffer. Then the cDNA fragments were purified with QiaQuick PCR extraction kit (Qiagen, Venlo, The Netherlands), end-repaired, added a base, and ligated to Illumina sequencing adapters. The ligation products were selected by agarose gel electrophoresis, PCR amplified, and sequenced using Illumina novaseq 6000 by Gene Denovo Biotechnology Co. (Guangzhou, China).

#### 2.6.2 Denovo assembly, unigene expression analysis and annotation

Reads obtained from the sequencing machines were further filtered by fastp (Chen et al., 2018) (version 0.18.0) to get high-quality clean reads. The parameters were removing reads containing adapters, more than 10% of unknown nucleotides (N) and more than 50% of low quality (Q-value ≤ 20) bases. Transcriptome denovo assembly was carried out with short reads assembling program-Trinity (Grabherr et al., 2011). The unigene expression was calculated and normalized to RPKM (Reads Per kb per Million reads) (Mortazavi et al., 2008). The formula is RPKM=(1000000*C)/(N*L/1000).C is the number of reads that are uniquely mapped to Unigene. N is the total number of reads that are uniquely mapped to all unigenes. L is the length (base number) of Unigene.

Then used BLASTx program (http://www.ncbi.nlm.nih.gov/BLAST/) with an E-value threshold of 1e-5 to NCBI non-redundant protein (Nr) database (http://www.ncbi.nlm.nih.gov), the Swiss-Prot protein database (http://www.expasy.ch/sprot), the Kyoto Encyclopedia of Genes and Genomes (KEGG) database (http://www.genome.jp/kegg), and the COG/KOG database (http://www.ncbi.nlm.nih.gov/COG). Protein functional annotations could then be obtained according to the best alignment results.

#### 2.6.3 Analysis of differentially expressed genes

RNAs differential expression analysis was performed by edgeR (Smyth, 2010). The genes with the parameter of false discovery rate (FDR)0.05 and absolute fold change≥2 were considered differentially expressed genes.

All DEGs were mapped to GO terms in the Gene Ontology database (http://www.geneontology.org/), gene numbers were calculated for every term, significantly enriched GO terms in DEGs comparing to the genome background were defined by hypergeometric test. The calculating formula of P-value is:


$$P=1-\sum_{\text{i}=0}^{\text{m-}1}\frac{\begin{array}{cc} \left(\begin{array}{c} M\\ \text{i} \end{array}\right) & \left(\begin{array}{c} N-M\\ \text{n-i} \end{array}\right) \end{array}}{\left(\begin{array}{c} N\\ \text{n} \end{array}\right)}$$

Here N is the number of all genes with GO annotation; n is the number of DEGs in N; M is the number of all genes that are annotated to the certain GO terms; m is the number of DEGs in M.

Comparing with the whole genome background, use Pathway enrichment analysis identified significantly enriched metabolic pathways or signal transduction pathways in DEGs. The calculating formula is the same as that in GO analysis.

#### 2.6.4 real-time quantitative PCR verification

Ten DEGs were randomly selected for real-time quantitative PCR (qRT-PCR), the selected DEGs names and primer information are shown in **Table 1**, refer to the specific method (Chen et al., 2022b).

**Table 1:** qRT-PCR Primer information.

Genes	Forward primer (5’ to 3’)	Reverse primer (5’ to 3’)
Unigene0006607	GACAACTAAGGGAGCAAGCG	AGGCCCTTCGATTTTGGAGA
Unigene0055110	TCTCCCACAGCCATCAAACT	CTGCCTGAACAATGCTCCAA
Unigene0074983	CTGGGACGTAAAGGGGATGT	CCCTAGTGTTCCCTGCTTCA
Unigene0073994	TAGTTCCGGCCATCCAACAA	TCGTATGTTCTGTCGCTGGA
Unigene0059768	CTGACTCAAACAGCAGCTCT	TTTTCTCCAACGTACGCAGC
Unigene0078694	GGTTTATGGCACCCTCATAGC	ACGAAAACCCATTGCAGACA
Unigene0068118	TCTCTACAGACAGGGTTGCC	CACACTTTGATTGGCGCTCT
Unigene0029314	GGCGAAAGGTTCTACACACG	GACGGTAAGTTCCAGTCCCA
Unigene0044913	TCTTCAGCTGCAAACCACAC	TTCGCAACTGAAGCTTTCCC
Unigene0075737	GGTGCTGTCGGATTAGGAGT	ATTCTTGTGGTCATCGGGGT

## 3 Result

### 3.1 Effects of different developmental stages and IAA concentrations on the germination of seed embryos after flowering

The results showed that the isolated embryos of different developmental stages could germinate on MS medium, but the germination rate varied greatly. The germination rate of young embryos at 70d was low, basically below 65%. With the continuous maturation of seed embryos, the germination rate of seed embryos gradually increased. In different developmental stages after flowering, except for 70d embryos, 80d, 90d, 100d, and 110d embryos had higher germination rates, all reaching more than 80%. Among them, 90d embryos had the highest germination rate, reaching 94.17% (**Table 2**).

**Table 2:** Peony embryo germination at different developmental stages.

Number	Seed age (d)	Basic medium	Number of explants	Germination rate (%)
1	70	MS	40	65 ± 4.08d
2	80	MS	40	86.67 ± 2.36b
3	90	MS	40	94.17 ± 1.18a
4	100	MS	40	87.5 ± 2.04b
5	110	MS	40	80.8 ± 1.18c

Different lowercase letters represent significant difference at 0.05 level (Duncan, P = 0.05).

### 3.2 Effects of different developmental stages on callus induction after flowering

**Figure 2:**
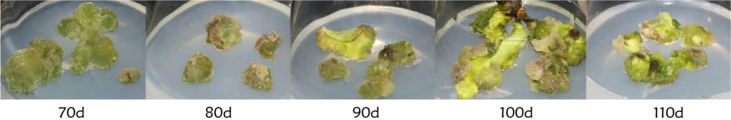
Induction of embryonic callus at different developmental stages.

It can be seen from **Table 3** that different developmental stages after flowering had a certain influence on the induction of callus from embryo cotyledons of “Fengdanbai”. Among them, the induction rate of 70d and 80d embryos was 100%. There was no significant difference in the induction rate of 70d, 80d and 90d embryos. The callus induction rate of 90d, 100d and 110d embryos gradually decreased, indicating that the seeds were in different developmental stages, the older the seedling age, the lower the callus induction rate. Cotyledon callus proliferation multiples of 80d and 90d were higher, reaching 5.31 and 4.77, respectively. At the same time, the cotyledons of seed embryos at different developmental stages were used as explants, and there were also great differences in their induction capacity. It is easier to induce callus from young embryos cultivated under the same conditions, and the time is also shorter. In the later growth process, the growth vigor and Proliferation multiples were better than those of mature seed embryos (**Figure 2**).

**Table 3:** Effects of different developmental stages on callus induction.

Seed age(d)	Number of explants	Callus induction (%)	Proliferation multiples
70	28	100.0 ± 0.00 a	4.19 ± 0.43 ab
80	28	100.0 ± 0.00 a	5.31 ± 0.67 c
90	28	98.81 ± 2.06 a	4.77 ± 0.37 bc
100	28	88.10 ± 4.12 b	3.95 ± 0.17 ab
110	28	86.25 ± 8.20 b	3.60 ± 0.42 a

Different lowercase letters represent significant difference at 0.05 level (Duncan, P = 0.05).

### 3.3 Effects of different plant growth regulators on callus induction of cotyledons in 80d developmental stage

**Figure 3:**
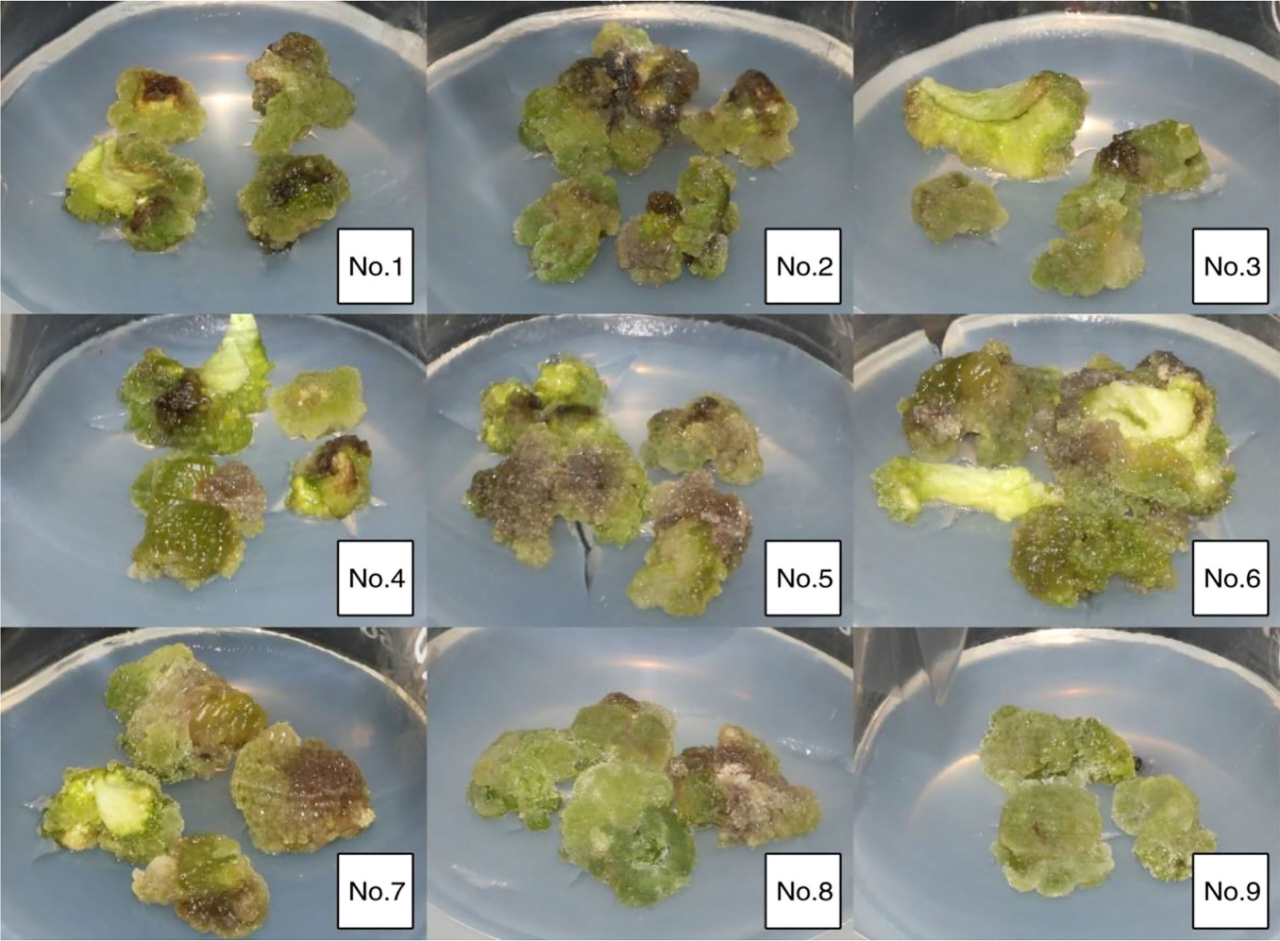
Growth of callus induced by cotyledons germinated from 80-day embryos *in vitro* on different media.

The 80d embryo-induced shoots were selected as explants, and the optimum medium for inducing callus was screened. It can be seen from **Table 4** that different concentrations of NAA and TDZ have significant differences in the induction of callus from embryo cotyledons of “Fengdanbai”. Among them, the induction rates of treatments 2, 3, 4, 5, 8, and 9 were significantly higher than those of other treatment groups, but there was no significant difference between these groups. The callus proliferation multiple of treatment 3 was the highest, which was 4.77, and there was a significant difference compared with other treatment groups. Considering the induction rate and multiplication factor, the optimal MS medium for inducing callus is 0.1 mg·L^–1^ NAA+0.3 mg·L^–1^ TDZ+3 mg·L^–1^ 2,4-D, and the induction rate can reach 98.81%; (**Figure 3**).

**Table 4:** Effects of different plant growth regulators on callus induction of cotyledons in 80d developmental stage.

Number	Basic medium	plant growth regulator (mg·L^–1^)			Number of explants	Frequency of callus induction (%)	Proliferationmultiples
		NAA	TDZ	2,4-D			
1	MS	0.1	0.1	3	28	80.95 ± 11.48 ab	1.79 ± 0.51 a
2	MS	0.1	0.2	3	28	89.29 ± 7.14 bc	2.65 ± 0.46 bc
3	MS	0.1	0.3	3	28	98.81 ± 2.06 c	4.77 ± 0.37 e
4	MS	0.3	0.1	3	28	88.10 ± 2.06 bc	2.46 ± 0.23 bc
5	MS	0.3	0.2	3	28	88.10 ± 5.46 bc	2.61 ± 0.26 bc
6	MS	0.3	0.3	3	28	77.38 ± 7.43 a	2.06 ± 0.29 ab
7	MS	0.5	0.1	3	28	71.43 ± 9.45 ab	2.04 ± 0.05 ab
8	MS	0.5	0.2	3	28	98.81 ± 2.06 c	2.89 ± 0.21 c
9	MS	0.5	0.3	3	28	98.81 ± 2.06 c	3.70 ± 0.55 d
CK	MS	0	0	0	28	72.62 ± 5.46 a	1.65 ± 0.10 a

Different lowercase letters represent significant difference at 0.05 level (Duncan, P = 0.05).

### 3.4 Analysis of morphological characteristics of different types of callus

**Figure 4:**
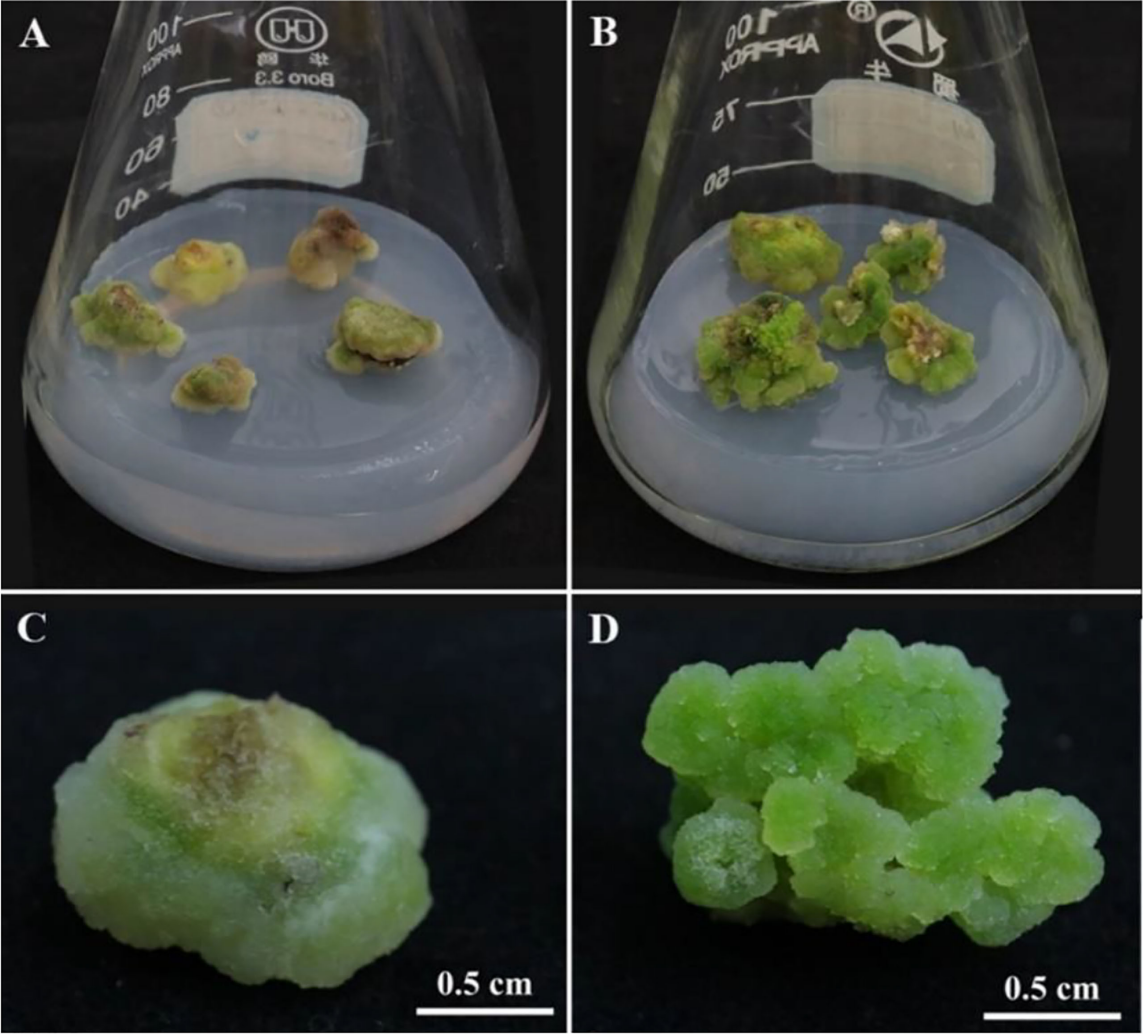
Morphology of callus with different differentiation potential. **(A, C)** The callus with low differentiation rate (LDR); **(B, D)** The callus with high differentiation rate (HDR).

In our experiment, two different types of callus were obtained by culturing under the same conditions (**Figure 4**). It can be seen from the figure that the callus of the peony seed embryo differentiated into two different colors of yellow-green and green, the yellow-green callus has a hard texture and a firm and smooth surface, as if it is wrapped by a layer of fine white material, which is relatively dry and has low moisture content (**Figures 4A, C**); The green callus is soft, crystal clear and translucent, with many small particles differentiated on the surface, the whole callus is dense and moist with high water content (**Figures 4B, D**). The differentiation potentials of these two types callus was significantly different, and the differentiation rate of green callus was significantly higher than that of yellow-green callus.

### 3.5 Quality control and statistics of transcriptome data of different morphological callus

The transcriptome data of different morphological callus were filtered for low-quality data, and the base quality was statistically analyzed (**Table 5**). After the reference samples were filtered, the Q20 quality of each sample was stable above 96%, the Q30 quality value was above 91%, and the GC content was stable above 44%, and the base quality was consistent with the subsequent analysis.

**Table 5:** Base quality statistics.

Sample	RawData (bp)	CleanData (bp)	Q20 (%)	Q30 (%)	N (%)	GC (%)
LDR-1	6234719100	6177586453	5978375290 (96.78%)	5657126820 (91.58%)	68072 (0.00%)	2753408492 (44.57%)
LDR-2	5971537500	5910157077	5708637843 (96.59%)	5390957124 (91.22%)	64101 (0.00%)	2634405421 (44.57%)
LDR-3	5868508500	5806664621	5610472480 (96.62%)	5300829324 (91.29%)	62693 (0.00%)	2590853135 (44.62%)
HDR-1	7026768000	6965857307	6749358949 (96.89%)	6395814079 (91.82%)	75684 (0.00%)	3119887512 (44.79%)
HDR-2	6424720500	6367384584	6163187409 (96.79%)	5832261744 (91.60%)	69037 (0.00%)	2845783393 (44.69%)
HDR-3	5994980700	5942932319	5758981344 (96.90%)	5457133810 (91.83%)	65255 (0.00%)	2656642177 (44.70%)

RawData (bp): Total number of off-machine data bases; CleanData (bp): The total number of high-quality data bases after filtering; Q20 (%): The number of bases with a quality value above Q20 and their percentage in CleanData; Q30 (%): The number of bases with a quality value above Q30 and their percentage in CleanData; N (%): the number of N bases in single-end reads and the percentage of CleanData; GC (%): GC ratio of filtered sequence bases.

**Figure 5:**
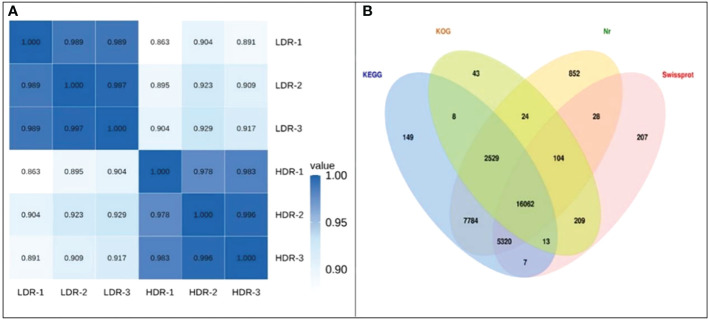
**(A)** Heatmap of Pearson correlation coefficients between replicates; **(B)** Venn diagram of the number of Unigenes annotated to the four major databases.

Take the expression levels in any two samples, calculate the Pearson correlation coefficients between each two samples, and then visually display the correlation between any two samples in the form of a heat map. As shown in the **Figure 5A**, the Pearson correlation coefficients between all samples are greater than 0.85, indicating that the repeatability between repeated samples within a group is good. In addition, **Figure 5B** shows the Venn diagram of the annotation results of all assembled Unigenes in the four major databases of Nr, SwissProt, KOG.

### 3.6 Analysis of differentially expressed genes in different morphological callus

**Figure 6:**
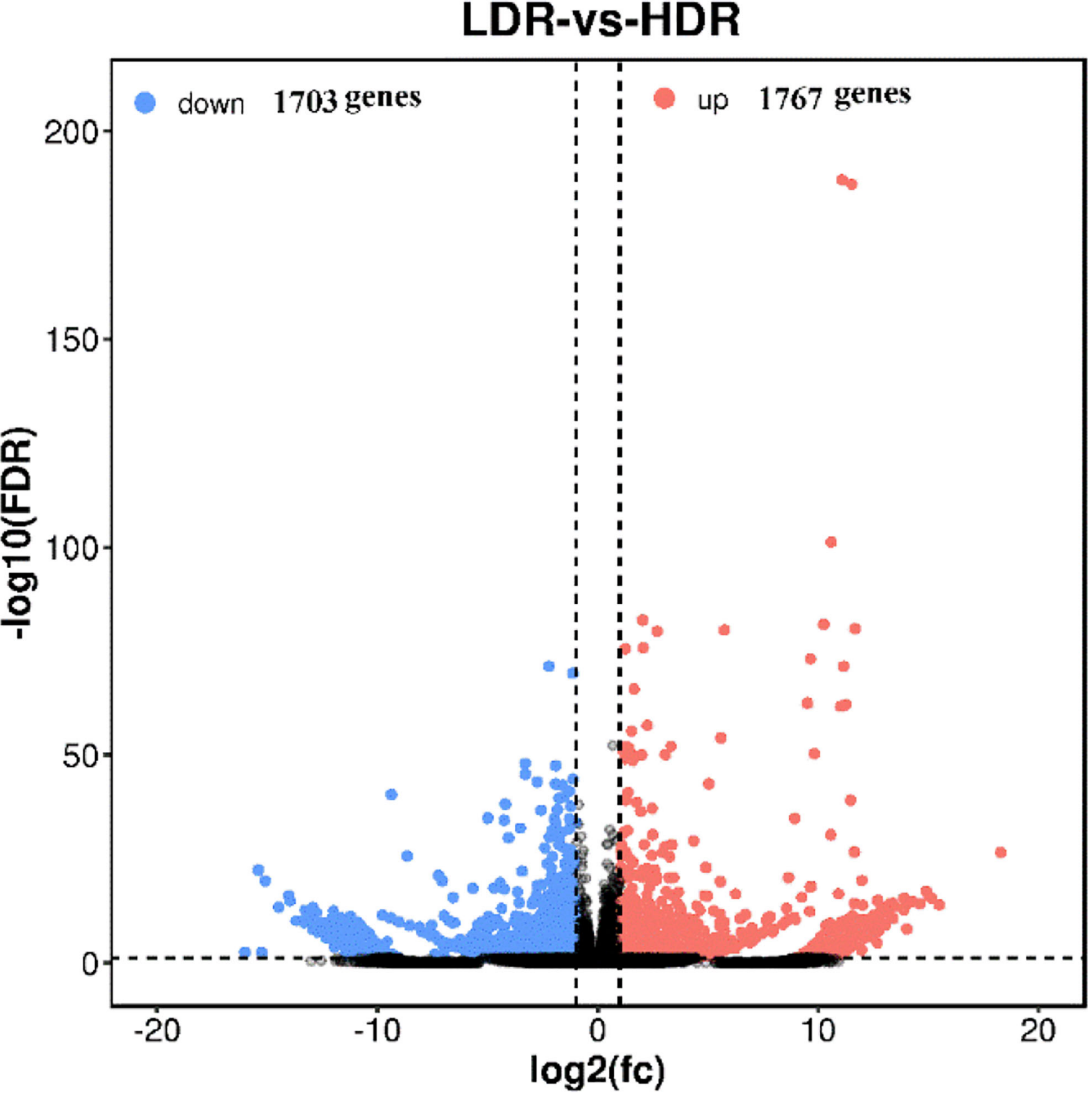
LDR vs HDR differentially expressed gene volcano plot The abscissa represents the logarithm of the difference between the two groups, and the ordinate represents the negative Log10 value of the FDR of the difference between the two groups. Blue dots indicate genes with down-regulated expression and red dots indicate genes with up-regulated expression.

The volcano plot comparing significantly differentially expressed genes between groups is used to visually display the difference genes between LDR and HDR. As shown in **Figure 6**, red (up-regulated) and blue (down-regulated) points indicate differences in gene expression, and black points indicate no difference, the closer the genes are to the two ends, the greater the degree of difference, among them, 1767 genes were significantly up-regulated and 1703 genes were significantly down-regulated between LDR and HDR groups.

### 3.7 GO enrichment analysis of differentially expressed genes in different morphological callus

**Figure 7:**
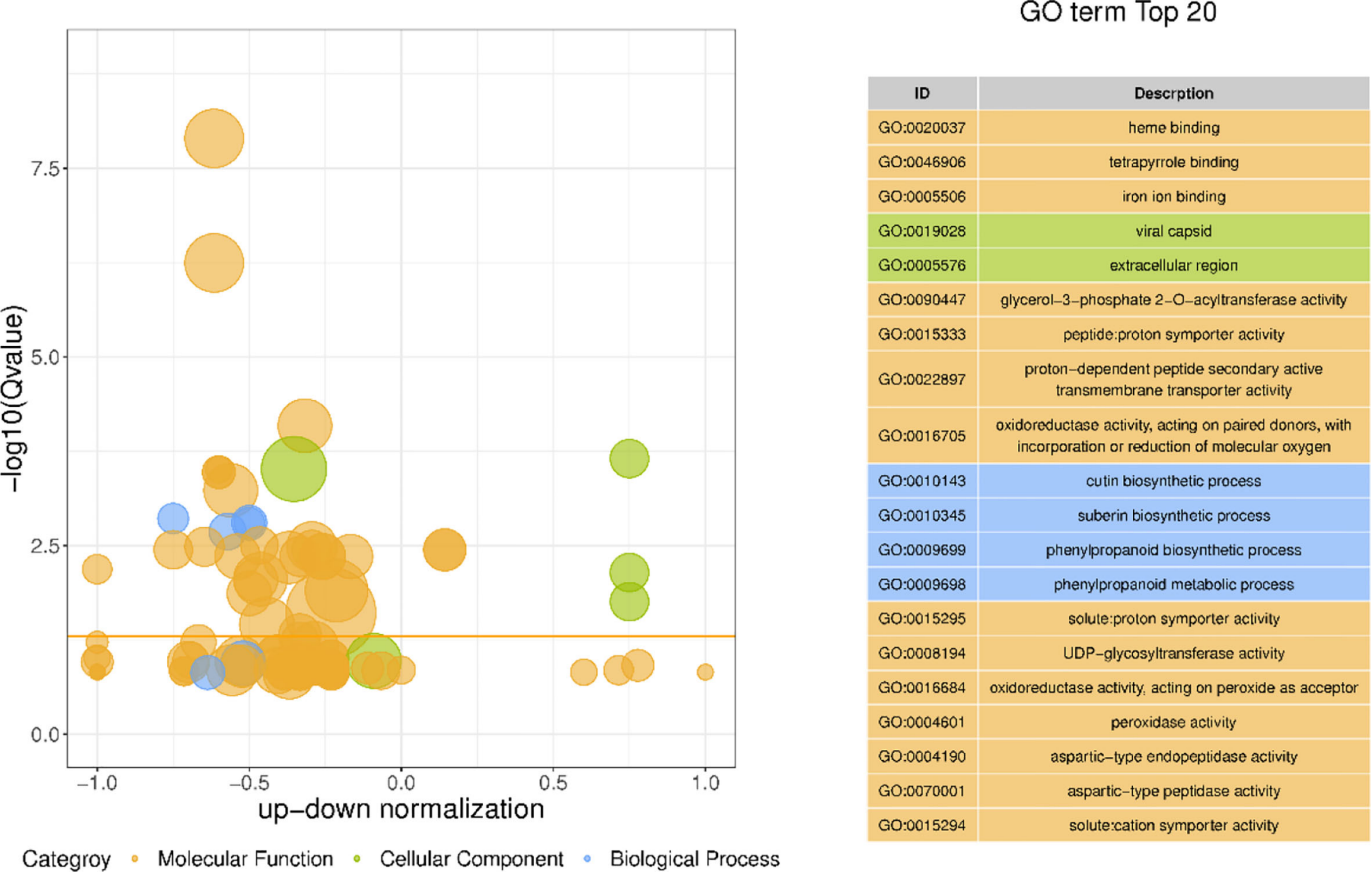
The bubble plot GO enrichment difference and top 20 items of GO enrichment of differentially expressed genes The ordinate is -log10 (Q value), the abscissa is the z-score value (the ratio of the difference between the number of up-regulated differential genes and the number of down-regulated differential genes to the total differential genes); the yellow line represents the threshold of Q value=0.05; On the right is a list of the top 20 GO terms with a Q value; different bubble colors and GO term colors in the figure represent different ontologies.

GO has a total of three ontologies, which describe the molecular function, fine cellular component, and biological process of genes respectively. Through the GO enrichment analysis of the differentially expressed genes in different morphological callus (**Figure 7**), the biological process GO analysis of these differentially expressed genes is mainly enriched in cutin biosynthetic process, suberin biosynthetic process, phenylpropanoid biosynthetic process, phenylpropanoid metabolic process, secondary metabolic process, etc., cell components are mainly enriched in viral capsid, extracellular region, virion part, virion, intrinsic component of plasma membrane, etc., and molecular functions are mainly enriched in heme binding, tetrapyrrole binding, iron ion binding, glycerol-3-phosphate 2-O-acyltransferase activity, peptide: proton symporter activity, etc.

### 3.8 KEGG enrichment analysis of differentially expressed genes in different morphological callus

**Figure 8:**
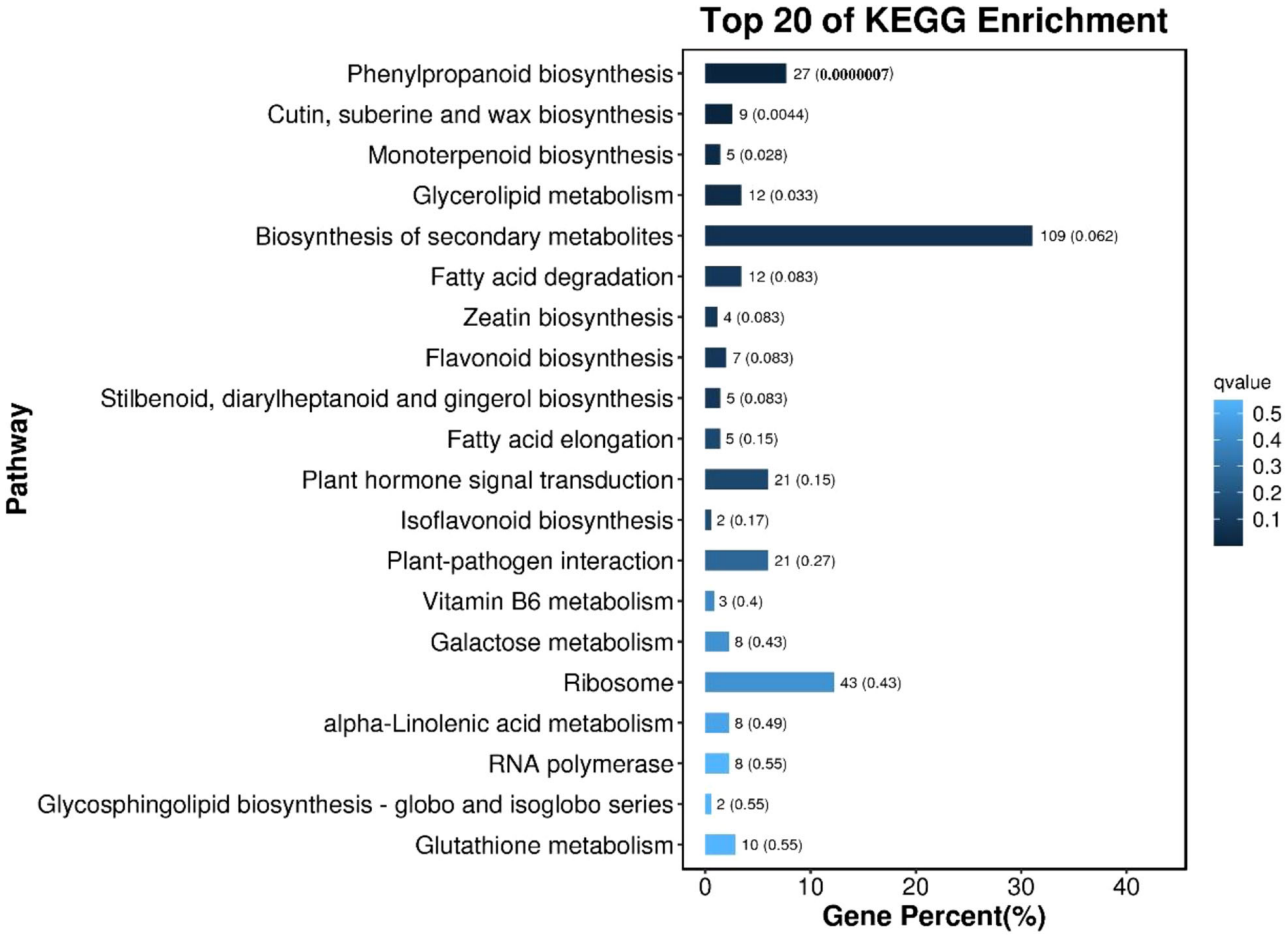
The bar chart of the top 20 KEGG enrichment pathways of differentially expressed genes The ordinate is the pathway, the abscissa is the percentage of the number of the pathway to the number of all differential genes, and the values on the column are the number of the pathway and the Q value.

KEGG enrichment pathway analysis was performed on the differentially expressed genes of callus with different morphological characteristics, as shown in **Figure 8**. The top 20 pathways with the smallest Q value were used to map, the darker the color, the smaller the Q value. The results showed that the differentially expressed genes in callus with high differentiation rate and low differentiation rate were mainly enriched in Phenylpropanoid biosynthesis; Cutin, suberine and wax biosynthesis; Monoterpenoid biosynthesis; Glycerolipid metabolism; Biosynthesis of secondary metabolites, etc.

### 3.9 Transcriptome data validation

**Figure 9:**
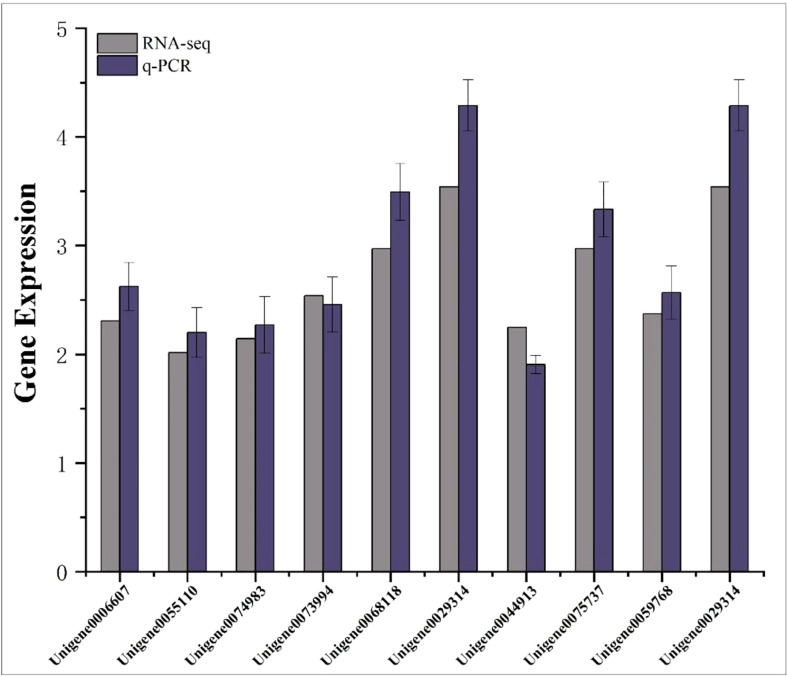
Transcriptome RPKM values and q-PCR relative expression values of differentially expressed genes. The abscissa represents the gene name; the ordinate represents the gene expression value.

Ten differentially expressed genes were randomly selected for qRT-PCR to verify the accuracy of RNA-seq results. The results showed that the relative expression trends of these genes were consistent with the gene expression trends of transcriptome sequencing (**Figure 9**), indicating that the transcriptome sequencing data were credible.

## 4 Discussion and conclusion

The induction and differentiation of peony callus is a crucial step in the establishment of the peony regeneration system. The type of explant, physiological state, and inoculation method have certain influence on the establishment of the regeneration system (Xu et al., 2017). There have been a lot of studies on explant selection, such as petal (Chen et al., 2022a), scale bud (Wang, 2016; Wang et al., 2018), stem segment (Zhu et al., 2015), anther (Liu et al., 2011), seed embryo (Zhang et al., 2011; Zhang et al., 2018) can be used as explants, among which the seed embryo has the highest viability and low contamination rate, which is an ideal material for inducing callus of peony (Lu et al., 2019), which is consistent with the results of this study. Nevertheless, peony seeds have typical epcotyl and hypocotyl dormancy characteristics (Cheng and Du, 2008), and seeds at different developmental stages can seriously affect the growth of *in vitro* embryos (He, 2006). Therefore, it is more feasible to select *in vitro* embryos from seeds of different developmental stages for culture, and chop the cotyledons of germinated *in vitro* embryos as explants to induce callus, which is further verified by the results of this study. According to the results of this study, the seed embryos of 80 d and 90 d have the best effect, the germination rate can be increased by more than 85%, and the callus induction rate can reach more than 98%, but the multiplication rate of the seed embryo of 80 d is higher. This is basically consistent with the results of previous studies (Yin et al., 2013; Yin et al., 2019; Lu et al., 2021), and is better than the results of Chen et al. (Chen et al., 2021) Using “Fengdanbai” mature embryos to directly induce callus, the highest induction rate is only 70.61%, and in our experimental results, the germination rate and callus induction rate of mature embryos were significantly lower than those of immature embryos in other periods, except for immature embryos whose germination rate was higher than 70 days. It is worth mentioning that although the germination rate and induction rate of mature embryos are not dominant compared to young embryos, the growth of embryos after germination is better, which may be due to the relatively sufficient nutrients accumulated in the mature embryos, resulting in large differences in the growth conditions at the early stage of germination, the specific reasons still need to be further explored. Overall, the 80-day seed embryo and its germinated cotyledons can be used as the best explants for callus induction of “Fengdanbai” peony. In addition, we also optimized the best medium for callus induction and proliferation using 80d seed embryos. Studies have shown that the induction of callus can be effectively achieved only when cytokinin and auxin are combined in a certain proportion (Wang et al., 2020). In this study, in addition to the common plant growth regulators NAA and 2,4-D, medium ratios of different concentrations of TDZ were also added. TDZ is a synthetic plant growth regulator with dual functions of cytokinin and auxin. It is more stable in plants and has higher activity than BA (Guo et al., 2005). The results showed that high concentration of TDZ combined with low concentration of NAA or high concentration of NAA could achieve better induction effect, but high concentration of TDZ combined with low concentration of NAA combined with induced callus had better multiplication multiples, which is consistent with the previous study that adding high concentrations of auxins and cytokinin plant growth regulators can significantly improve the callus induction rate of ‘Fengdanbai’ (Chen et al., 2021). So it is considered that the optimal medium combination is MS+0.1 mg·L^–1^NAA+0.3 mg·L^–1^TDZ+3 mg·L^–1^2,4-D.

The structural characteristics of callus can significantly affect its differentiation (Du et al., 2020). In our previous study, we take the peony petals as explants for callus induction, observed with different bud differentiation rate of callus morphology and ultrastructure, and found that there were great differences in their morphology and surface structure, selection of callus with closely arranged protrusions on the surface can improve the differentiation efficiency of callus (Chen et al., 2022a), the results were consistent with this study, which further demonstrated that the structure and morphology of callus could be used as a basis for screening the differentiation potential of callus. In the study of the molecular mechanism of callus differentiation, we conducted a transcriptomic comparative analysis of callus with different differentiation potentials, and the results showed that there were 3470 gene differences in callus with high differentiation rate and low differentiation rate, these genes may be the key genes responsible for the differences in their differentiation rates. GO and KEGG enrichment analysis was performed on these differentially expressed genes, and similar results were obtained. The KEGG metabolic pathway enrichment analysis showed that these differentially expressed genes were mainly enriched in the “Phenylpropanoid biosynthesis” metabolic pathway, which had a total of 27 genes were differentially expressed, of which 6 were up-regulated and 21 were down-regulated, including *PAL, PER, CCR, 4CL, HOMT* and other related genes. Studies have shown that the *PAL* gene is up-regulated in browned ginkgo callus, resulting in the increase of secondary metabolites and the accumulation of phenolic substances, which may lead to the browning of the callus (Solecka and Kacperska, 2003; Xu et al., 2021), and the up-regulation of *CCR* gene can increase the metabolism of phenolic acids such as coumaric acid and caffeic acid, which will lead to the accumulation of aldehydes and browning of callus (Xu et al., 2021). In this study, most of the genes in the “Phenylpropanoid biosynthesis” metabolic pathway were significantly down-regulated in callus with high differentiation rate compared with callus with low differentiation rate, including the above-mentioned *PAL* (*Unigene0070017*) and *CCR2* (*Unigene0003759*) genes, indicating that the callus with low differentiation rate may be due to the up-regulated expression of some genes in the phenylpropane metabolic pathway leading to its browning, which in turn affects its further differentiation, which is consistent with the phenomenon we observed in subsequent experiments, callus with low differentiation rate gradually browned seriously in further differentiation culture. We also found that two peroxidase genes were significantly down-regulated in callus with high differentiation rate relative to callus with low differentiation rate in this pathway, namely *Unigene0015233* (*PER62*) and *Unigene0015235* (*poxN1*). Yang et al. (2018) compared the properties of peroxidase isozymes in the callus of salt-tolerant mutants of alfalfa and ordinary callus at two growth stages and found that the type and content of peroxidase in salt-tolerant alfalfa mutant were reduced compared with that of wild type, indicating that it was related to plant stress resistance, which to a certain extent can help callus to improve the ability to resist microbial infection to quickly adapt to the environment of the differentiation medium. In addition, the second enrichment pathway of these differentially expressed genes is “cutin, suberin, and wax biosynthesis”. Cutin and lignin formed by this metabolic pathway can effectively limit plant water loss and improve mechanical strength to resist microorganisms, which may be one of the reasons why the callus with high differentiation rate looks wetter as a whole.

## Data availability statement

The data presented in the study are deposited in the GSA database of CNCB repository, accession number CRA008510.

## Author contributions

XC and XZ planned and designed the research. XZ, HZ, EH, ZL and BL participated in the tissue culture of embryo germination, callus induction and proliferation. XZ, XC, and WJ were responsible for mapping of transcriptome data and data analysis. XC and XZ were involved in manuscript editing. All authors contributed to the article and approved the submitted version.

## Funding

This research was funded by the National Key R&D Program of China, Grant Number 2019YFD1001500; the Basic Public Welfare Research Project in Zhejiang Province, Grant Number LGN22C160006 and the Talent Project of Jiyang College of Zhejiang A&F University, Grant Number RQ2020B04/RQ1911B05.

## Acknowledgments

We are grateful to Gene Denovo Biotechnology Co.,Ltd. (Guangzhou, China) for assisting in transcriptome sequencing.

## Conflict of interest

The authors declare that the research was conducted in the absence of any commercial or financial relationships that could be construed as a potential conflict of interest.

## Publisher’s note

All claims expressed in this article are solely those of the authors and do not necessarily represent those of their affiliated organizations, or those of the publisher, the editors and the reviewers. Any product that may be evaluated in this article, or claim that may be made by its manufacturer, is not guaranteed or endorsed by the publisher.
